# A Network Analysis of Panic Disorder, Agoraphobia, and Generalized Anxiety Disorder in 463 Patients From a Psychiatric Hospital

**DOI:** 10.1002/brb3.71241

**Published:** 2026-02-10

**Authors:** Emanuela Pizzolla, Juan Martin Tecco, Moritz Bruno Petzold, Giovanni Briganti

**Affiliations:** ^1^ Department of Computational Medicine and Neuropsychiatry, Faculty of Medicine University of Mons Mons Belgium; ^2^ CHUP‐MB, Department of Adult and Child Psychiatry Mons Belgium; ^3^ Department of Psychology Medical School Berlin Berlin Germany

**Keywords:** anxiety disorders, comorbidity, gad, network analysis, psychiatry

## Abstract

**Introduction:**

Panic disorder, agoraphobia, and generalized anxiety disorder (GAD) frequently co‐occur and share overlapping symptoms, yet it remains unclear whether they reflect distinct or interconnected symptom systems. This study examined the network structure of these disorders using clinician‐administered diagnostic data.

**Methods:**

A total of 463 adults completed the Mini International Neuropsychiatric Interview (M.I.N.I.) conducted by trained clinicians. Eighteen items from the panic disorder, agoraphobia, and GAD modules were retained after a multistep selection procedure ensuring clinical relevance, endorsement variability, and nonredundancy. A binary Ising network was estimated using eLASSO with EBIC model selection. Network accuracy, stability, and edge differences were evaluated through nonparametric bootstrapping.

**Results:**

The estimated network revealed two well‐defined symptom clusters corresponding to (1) panic–agoraphobia and (2) GAD. Within the panic–agoraphobia cluster, physiological symptoms (e.g., palpitations, shortness of breath, sweating, dizziness) were tightly interconnected, and catastrophic cognitions (fear of dying, fear of losing control) were moderately linked to bodily sensations. Agoraphobia symptoms were strongly connected to each other but relatively peripheral to other panic symptoms. The GAD cluster was anchored by difficulty controlling worry, which emerged as the most central symptom and showed strong associations with restlessness, sleep disturbance, fatigue, and irritability. Notably, no direct edges were found between panic–agoraphobia and GAD symptoms, suggesting distinct anxiety systems.

**Conclusion:**

These findings indicate that fear‐based and worry‐based anxiety symptoms form separable yet clinically relevant structures. Focusing on core processes like excessive worry and interoceptive regulation could enhance the specificity of interventions and more effectively disrupt anxiety maintenance mechanisms.

## Graphical abstract

Network analysis of clinician‐administered diagnostic data reveals two distinct anxiety symptom systems: a panic–agoraphobia network dominated by interconnected physiological symptoms and a generalized anxiety disorder network structured around cognitive worry processes, underscoring divergent fear‐based and worry‐based mechanisms within anxiety psychopathology.

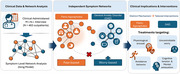

## 1 Introduction

Anxiety disorders represent a leading cause of disability worldwide, with lifetime prevalence estimates exceeding 25% in the general population (Kessler et al. 2005; WHO 2017). Among these conditions, panic disorder, agoraphobia, and generalized anxiety disorder (GAD) are particularly burdensome, often emerging early in life, running a chronic course, and severely impairing social, occupational, and personal functioning (Kessler et al. 2006; Wittchen and Jacobi 2005).

Panic disorder is characterized by recurrent episodes of sudden, intense fear accompanied by physiological arousal and catastrophic cognitions, while agoraphobia involves avoidance of situations in which escape might be difficult in the event of panic‐like symptoms (American Psychiatric Association 2013; Barlow 2002). In contrast, GAD is defined by excessive, uncontrollable worry coupled with chronic tension, irritability, fatigue, and sleep disturbance (Ruscio et al. 2017). Despite these nosological distinctions, clinical comorbidity between panic, agoraphobia, and GAD is strikingly high, with many patients meeting criteria for multiple anxiety disorders over their lifetime (Ruscio et al. 2007; Grant et al. 2005; Wittchen et al. 2010). This overlap raises fundamental questions about whether current diagnostic systems capture the true architecture of anxiety psychopathology.

Traditional frameworks such as the Diagnostic and Statistical Manual of Mental Disorders (DSM‐5) and the International Classification of Diseases (ICD‐11) conceptualize mental disorders as latent entities reflected by sets of interchangeable symptoms. Within this view, symptoms are treated as passive indicators of an underlying condition rather than as active components of psychopathology. However, patients with the same diagnosis can show markedly different symptom profiles, and cross‐cutting features such as sleep disturbance, irritability, and avoidance appear in multiple syndromes, making it difficult to explain high comorbidity or identify symptoms most relevant to maintenance and treatment (Fried and Cramer 2017; Dalgleish et al. 2020; Krueger and Bezdjian 2009). These limitations have prompted a shift toward dimensional and transdiagnostic approaches, emphasizing the role of individual symptoms and their interactions in shaping psychopathology.

Network analysis advances this perspective by conceptualizing mental disorders not as latent diseases but as systems of interacting symptoms, modeled as networks in which symptoms are nodes connected by edges representing statistical dependencies (Borsboom 2017; Borsboom and Cramer 2013). From this standpoint, psychopathology emerges from the dynamic interplay among symptoms, allowing identification of “central” symptoms that sustain the network and “bridge” symptoms that connect otherwise distinct clusters, thereby offering a mechanistic account of both within‐disorder coherence and cross‐disorder comorbidity (McNally 2016; Robinaugh et al. 2020). Importantly, DSM‐5–based diagnostic frameworks and network analysis operate at different explanatory levels: the former provides a categorical system for classification and clinical communication, whereas the latter offers a symptom‐level model aimed at understanding patterns of symptom co‐occurrence and potential maintenance mechanisms. Accordingly, findings derived from network analysis should not be interpreted as challenging diagnostic categories, but rather as characterizing the structure and interrelations of symptoms within a specific population.

Network and clustering studies of anxiety disorders have provided important insights into how symptoms interact. In panic disorder, symptoms often organize into groups reflecting physiological arousal (e.g., dyspnea, choking, sweating, nausea), catastrophic fears (e.g., fear of dying, fear of going crazy), and anticipatory anxiety/agoraphobia (Shioiri et al. 1996), with avoidance bridging these anticipatory fears and core panic symptoms (Cox et al. 1991; Kim et al. 2025). Research on GAD, by contrast, has consistently shown that cognitive features, particularly excessive worry and fear of losing control, emerge as central nodes, anchoring associations with a broad range of somatic and emotional symptoms (Beard et al. 2016; Heeren et al. 2021; Zhang et al. 2024). Despite these insights, important gaps remain. Most prior studies have examined single disorders rather than modeling panic, agoraphobia, and GAD symptoms within the same population, leaving unclear whether they form distinct communities or share bridging mechanisms. Moreover, the common reliance on self‐report questionnaires (e.g., GAD‐7, Panic Disorder Severity Scale) may limit diagnostic precision and generalizability compared to clinician‐administered interviews (Heeren and McNally 2016; Robinaugh et al. 2020).

The present study addresses these gaps by applying network analysis to a large clinical sample assessed with the Mini International Neuropsychiatric Interview (M.I.N.I.; Sheehan et al. 1998). The interview was administered by trained clinicians to enhance diagnostic precision and reduce biases typical of unsupervised self‐report measures (Bergelson et al. 2022; Rosenman et al. 2011). Within this framework, we focused on the panic disorder, agoraphobia, and GAD modules, selecting a refined, nonredundant set of clinically relevant items to capture distinct and temporally specific symptoms.

Our objectives were threefold: (1) to chart the symptom‐level network structure of panic disorder, agoraphobia, and GAD in a clinically assessed sample; (2) to identify central symptoms that may serve as effective intervention targets; and (3) to examine potential bridge symptoms that help explain comorbidity across these anxiety conditions. By combining structured diagnostic interviews with contemporary network‐analytic methods, we aim to advance a symptom‐level understanding of anxiety architecture that moves beyond traditional categorical boundaries.

## 2 Method

### 2.1 Participants

We analyzed data from 463 adult outpatients (F: 309, M: 154; age, M ± SD = 49.94 ± 12.74, range: 20–98 years). Participants in our study were primarily referred by nonpsychiatrist physicians for diagnostic evaluation due to persistent or debilitating anxiety‐related symptoms, as identified by their referring physicians. Common referral reasons included: suspected GAD, symptoms suggestive of Panic Disorder or Agoraphobia, or unclear diagnostic presentations requiring specialized assessment.

Participants underwent the M.I.N.I. (French version 5.0.0; Sheehan et al. 1998) between January 2007 and September 2020 as part of a psychiatric evaluation. Assessments were carried out at the Centre Hospitalier Psychiatrique (CHP) “Le Chêne aux Haies” in Mons, Belgium. All data were fully anonymized prior to analysis, and the study protocol received approval from the CHP Ethics Committee (accreditation number: 951; approval date: June 24, 2024).

To be included, participants had to be at least 18 years old, fluent in French, and have completed the anxiety‐related modules of the M.I.N.I.: Module E (Panic Disorder), Module F (Agoraphobia), and Module O (GAD). No restrictions were applied regarding whether individuals met full DSM‐IV diagnostic thresholds, since the aim was to explore symptom‐level associations rather than categorical diagnoses. As a result, the sample comprised a clinically heterogeneous population, including individuals meeting full DSM‐IV diagnostic criteria as well as those presenting with subthreshold. An overview of diagnostic endorsement across M.I.N.I. dimensions in the sample is provided in Table 1.

**TABLE 1:** Diagnostic endorsement for each M.I.N.I. dimensions.

Diagnostic dimension	Criteria Met N (%)	Criteria not Met N (%)
Major depressive episode (MDE)	Current: 411 (88.8)	52 (11.2)
Dysthymia	Current: 38 (8.2) Past: 1 (0.2)	424 (91.6)
Suicidality	Low: 123 (26.6) Moderate: 39 (8.4) High: 163 (35.2)	138 (29.8)
(Hypo)manic episode	Hypomanic current: 26 (5.6) Hypomanic past: 24 (5.2) Manic current: 27 (5.8) Manic past: 76 (16.4)	332 (71.7)
Panic disorder	Current: 128 (27.6) Past: 28 (6.0)	307 (66.3)
Agoraphobia	Current: 172 (37.1)	291 (62.9)
Social phobia	Current: 79 (17.1) Past: 12 (2.6)	372 (80.3)
Obsessive–compulsive disorder (OCD)	Current: 72 (15.6)	391 (84.4)
Post‐traumatic stress disorder (PTSD)	Current: 53 (11.4)	410 (88.6)
Alcohol use disorder	Dependence: 62 (13.4) Abuse: 5 (1.1)	401 (86.6)
Substance use disorder (non‐alcohol)	Dependence: 27 (5.8) Abuse: 4 (0.9)	436 (94.2)
Psychotic disorders	Mood disorder with psychotic features: Current: 62 (13.4) Past: 129 (27.9) Psychotic syndrome: Current: 61 (13.2) Past: 43 (9.3)	334 (72.2)
Anorexia nervosa	Current: 2 (0.4)	461 (99.6)
Bulimia nervosa	Current: 26 (5.6)	437 (94.4)
Generalized anxiety disorder (GAD)	Current: 224 (48.4)	239 (51.6)

*Note*. Percentages are calculated relative to the total sample (*N* = 463). Diagnoses are based on categorical DSM‐IV criteria assessed with the M.I.N.I. Subcategories are reported where available. This table describes diagnostic endorsement only and does not reflect symptom severity, functional impairment, or formal comorbidity rates.

### 2.2 Measurement

Psychopathological assessment was conducted using the M.I.N.I., French version, a brief, structured diagnostic interview that assesses DSM‐IV Axis I disorders using dichotomous (yes/no) items, and shows good concordance with longer instruments such as the SCID and CIDI (Lecrubier et al. 1997; Sheehan et al. 1998). The M.I.N.I. systematically evaluates whether participants meet the DSM‐IV criteria for specific disorders, but does not provide a definitive clinical diagnosis on its own or indices of symptom severity.

In the current study, three modules were included: Module E (panic disorder), Module F (agoraphobia), and Module O (GAD) (Table 2). Rather than including all items from each module, we selected a subset of clinically and theoretically relevant symptoms based on a multistep procedure emphasizing clinical interpretability, temporal relevance, and network parsimony.

**TABLE 2:** Descriptive overview of candidate items from the M.I.N.I. modules assessing panic disorder, agoraphobia, and generalized anxiety disorder.

Item code	Diagnostic category	Brief description	Endorsement (%)
E1	Panic disorder	Sudden panic attacks	64.36
E2	Panic disorder	Unexpected panic spells	48.38
E3	Panic disorder	Persistent fear after panic	36.93
E4a	Panic disorder	Racing or pounding heart	33.69
E4b	Panic disorder	Sweating or clammy hands	29.81
E4c	Panic disorder	Trembling or shaking	27.86
E4d	Panic disorder	Shortness of breath	33.05
E4e	Panic disorder	Choking sensation	31.10
E4f	Panic disorder	Chest pain or discomfort	29.16
E4g	Panic disorder	Nausea or stomach problems	21.81
E4h	Panic disorder	Dizziness or lightheadedness	27.86
E4i	Panic disorder	Feelings of unreality or detachment	18.79
E4j	Panic disorder	Fear of losing control or going crazy	29.37
E4k	Panic disorder	Fear of dying	22.25
E4l	Panic disorder	Tingling or numbness	20.30
E4m	Panic disorder	Hot flashes or chills	17.21
E6	Panic disorder	Recurrent panic attacks	29.37
F1	Agoraphobia	Anxiety in public or difficult‐to‐escape places	41.90
F2	Agoraphobia	Avoidance or need for companion	37.15
O1a	GAD	Excessive daily worry	68.03
O1b	GAD	Worries present most days	59.61
O2	GAD	Difficulty controlling worry	50.54
O3a	GAD	Restlessness	46.22
O3b	GAD	Feeling tense	41.68
O3c	GAD	Fatigue	43.84
O3d	GAD	Difficulty concentrating	42.76
O3e	GAD	Irritability	39.52
O3f	GAD	Sleep disturbance	39.96

*Note*: This table lists all 28 symptom items considered for inclusion in the network analysis, organized by diagnostic category. Each item is identified by its original M.I.N.I. code, a brief symptom description, and the percentage of participants endorsing the symptom (yes = 1).

Items assessing overlapping constructs, such as E1 and E3, which reference persistent panic‐related fear, were excluded in favor of more temporally specific alternatives (e.g., E6, which targets recent panic symptoms). Second, items were screened based on endorsement rates (Table 2). Endorsement frequency was used as an auxiliary criterion to evaluate item informativeness, considering too low‐ or high‐frequency endorsement is considered an indicator of reduced informativeness and potential unreliability in network estimation (Rodebaugh et al. 2018). The whole pull items had endorsement rates ranging approximately from 17% to 68%.

To further reduce redundancy, we applied a standardized implementation of the goldbricker method, which identifies item pairs with more than 75% shared correlation profiles across the dataset (Jones 2017, 2024). Items flagged as statistically redundant were reviewed in terms of content, variability, and clinical relevance, and only one representative item was retained per redundant pair. The complete item selection process, including the list of excluded items (Table S1) and the corresponding redundancy heatmaps (Figure S1), is reported in the Supporting Information.

This process resulted in a final set of 18 items that were conceptually distinct, temporally specific, and statistically nonredundant (Table 3). For readability and clarity in network visualizations, variable labels were standardized (Table 3).

**TABLE 3:** Final set of symptom items included in the network analysis.

Item code	MINI question	Network label
E4a	Did you have skipping, racing or pounding of your heart?	Fast_HB
E4b	Did you have sweating or clammy hands?	Sweat
E4c	Were you trembling or shaking?	Tremble
E4d	Did you have shortness of breath or difficulty breathing?	BreathDiff
E4g	Did you have nausea, stomach problems or sudden diarrhea?	Stomach
E4h	Did you feel dizzy, unsteady, lightheaded or faint?	LightHead
E4j	Did you fear that you were losing control or going crazy?	FearCtrl
E4k	Did you fear that you were dying?	FearDie
E6	In the past month, did you have attacks when you suddenly felt anxious, frightened, uncomfortable or uneasy, even in situations where most people would not feel that way repeatedly (2 or more) followed by persistent fear of having another attack? *	Panic
F1	Do you feel anxious or particularly uneasy in places or situations from which escape might be difficult, and where help might not be available in case of panic attack, like being in a crowd, standing in a line (queue), when you are alone away from home or alone at home, or when crossing a bridge, traveling in a bus, train or car?	Ago_Anx
F2	Do you fear these situations so much that you avoid them, or suffer through them, or need a companion to face them?	Ago_Avoid
O1b	Are you worried excessively or been anxious about several things of day to day life most days? *	Worry
O2	Do you find it difficult to control the worries or do they interfere with your ability to focus?	WorryCtrl
O3a	Feel restless, keyed up or on edge?	Restless
O3c	Feel tired, weak or exhausted easily?	Fatigue
O3d	Have difficulty concentrating or find your mind going blank?	FocusDiff
O3e	Feel irritable?	Irritability
O3f	Have difficulty sleeping (falling asleep, waking up, early wakening)?	SleepDiff

*Note*: Each row presents the original M.I.N.I. item code, the full question as worded in the French version (translated into English), and the abbreviated label used in the network visualization. Items marked with an asterisk (*) were reworded slightly to enhance clarity and maintain semantic integrity while improving standalone interpretability within the network context.

### 2.3 Network Analysis

Network analyses were conducted in R software (version 3.5.1), an open‐source statistical programming environment (https://www.r‐project.org/), using packages previously applied in related studies from the same laboratory (e.g., Briganti and Linkowski 2020). The core packages used for network estimation and visualization were *qgraph* (Epskamp et al. 2012) and *IsingFit* (van Borkulo et al. 2014), while the *bootnet* package (Epskamp, Borsboom, et al. 2018) was used for estimating the stability and accuracy of the network structure.

### 2.4 Network Estimation

We estimated a binary symptom network using the Ising model (IsingFit algorithm) implemented in *bootnet* (van Borkulo et al. 2014). This approach applies L1‐regularized logistic regression with EBIC model selection (eLASSO; Epskamp and Fried 2018; Foygel and Drton 2010), retaining only the most stable conditional associations among dichotomous items. The tuning parameter γ was set to 0.25 (van Borkulo et al. 2014), following standard practice in psychiatric network analysis (Briganti and Linkowski 2020; Goodwin et al. 2023).

The final network was visualized using a Fruchterman–Reingold layout (Fruchterman and Reingold 1991), where each symptom is represented as a node and edges reflect regularized partial correlations between pairs of symptoms after controlling for all other symptoms in the network. Edge weights quantify this association, with larger absolute values, represented by greater edge thickness, indicating stronger conditional associations. Only edges surviving regularization are displayed, yielding a sparse and interpretable structure, but their magnitude is model‐dependent and primarily meaningful in relative terms within the same network.

### 2.5 Network Inference

To investigate the relative importance of each node in the network, we computed centrality indices (Boccaletti et al. 2006) using the *centrality* function from the qgraph package (Epskamp et al. 2012). Our primary focus was on strength centrality, which quantifies the degree to which a node is directly connected to others in the network. Specifically, it represents the sum of the absolute weights of all edges connected to a given node, providing an index of how strongly a symptom is interconnected with the rest of the network (Boccaletti et al. 2006; Briganti et al. 2024). A centrality plot was generated to visualize the relative strength of each item across the network.

### 2.6 Network Stability

We assessed network accuracy and stability using nonparametric bootstrapping with 2,000 resamples in the *bootnet* package (Briganti and Linkowski 2020; Epskamp, Borsboom, et al. 2018). Edge‐weight bootstraps provided confidence intervals (CIs) for each association and allowed comparison of edge strengths. Stability of strength centrality was evaluated via case‐dropping bootstraps, yielding the centrality–stability (CS) coefficient, which reflects how much of the sample can be removed while preserving centrality estimates. Values above 0.50 are considered sufficient for reliable interpretation, while values below 0.25 indicate instability (Epskamp, Borsboom, et al. 2018). We also conducted centrality difference tests to examine whether nodes significantly differed in strength. These procedures offered a comprehensive assessment of network accuracy and robustness, following current best practices in psychological network analysis (Epskamp, Borsboom, et al. 2018; Briganti et al. 2024).

## 3 Results

### 3.1 Network Estimation

**FIGURE 1:**
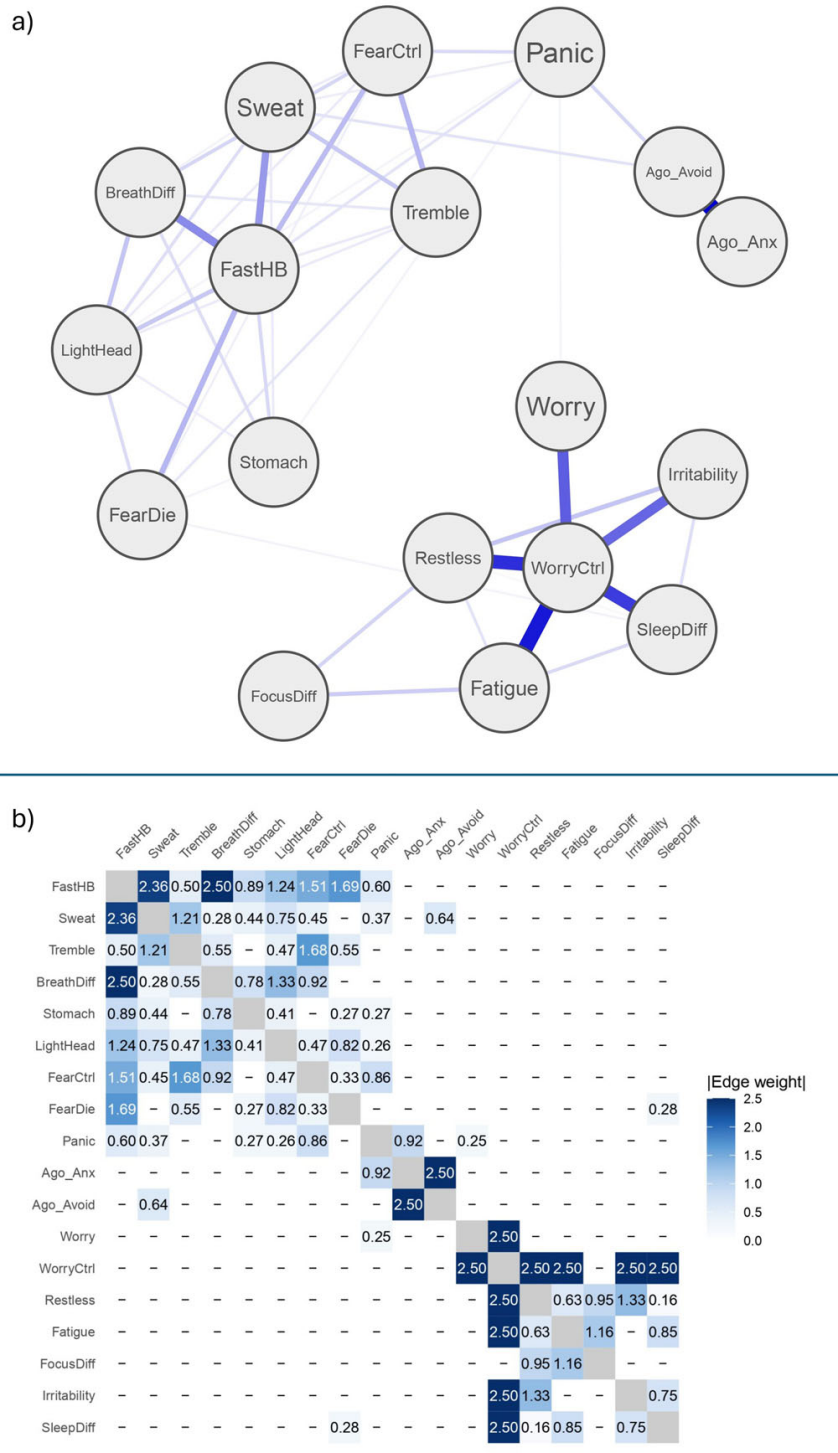
Estimated network structure and edge‐weight matrix for symptoms of Panic Disorder, Agoraphobia, and GAD. (a) IsingFit network showing two main clusters: a panic–agoraphobia cluster (top) and a GAD cluster (bottom). Thicker edges indicate stronger conditional associations. (b) Heatmap of regularized edge weights (range = 0.25–2.50), with darker blue denoting stronger connections.

The estimated network structure is shown in Figure 1a, with the corresponding edge‐weight matrix in Figure 1b. The IsingFit model retained only the most stable conditional associations between symptoms.

Two primary clusters emerged: one comprising Panic Disorder and Agoraphobia symptoms, and another reflecting GAD‐related features. Within the panic cluster, physiological symptoms such as heart racing (*Fast_HB*), shortness of breath (*BreathDiff*), excessive sweat (*Sweat*), trembling (*Tremble*), and dizziness (*LightHead*) were strongly interconnected, with the strongest edges linking *Fast_HB–Tremble* (edge weight = 2.50), *Fast_HB–Sweat* (= 2.36), and *Tremble–BreathDiff* (= 1.33), forming a dense autonomic‐arousal subnetwork. Cognitive symptoms like fear of losing control (*FearCtrl*) and fear of dying (*FearDie*) were moderately linked to these bodily sensations, particularly *Fast_HB* (= 0.78) and *Tremble* (= 0.47). *Ago_Avoid* and *Ago_Anx* formed one of the strongest connections (= 2.50), consistent with the co‐occurrence of anticipatory fear and avoidance behavior (Barlow 2002) and showed modest links to panic symptoms such as *Panic* (= 0.95).

The second cluster consisted of GAD‐related symptoms anchored around difficulty controlling worry (*WorryCtrl*). This node showed strong connections to *Restlessness* (= 1.33), *Sleep problems* (= 0.85), *Fatigue* (= 0.75), and *Irritability* (= 0.75), indicating persistent physiological and cognitive tension associated with chronic worry. However, despite their strong links to WorryCtrl, these symptoms were only weakly connected to one another. In particular, the weak *Fatigue–SleepDiff* link suggests a relative dissociation between low energy and sleep disturbance in this sample.

Overall, no strong direct edges were observed between the panic–agoraphobia and GAD clusters, indicating that these dimensions were largely independent. The resulting structure formed two coherent groups consistent with DSM‐based distinctions between fear‐based disorders, like panic and agoraphobia, marked by autonomic arousal and avoidance, and worry‐based disorders such as GAD, characterized by persistent cognitive tension.

### 3.2 Network Inference

**FIGURE 2:**
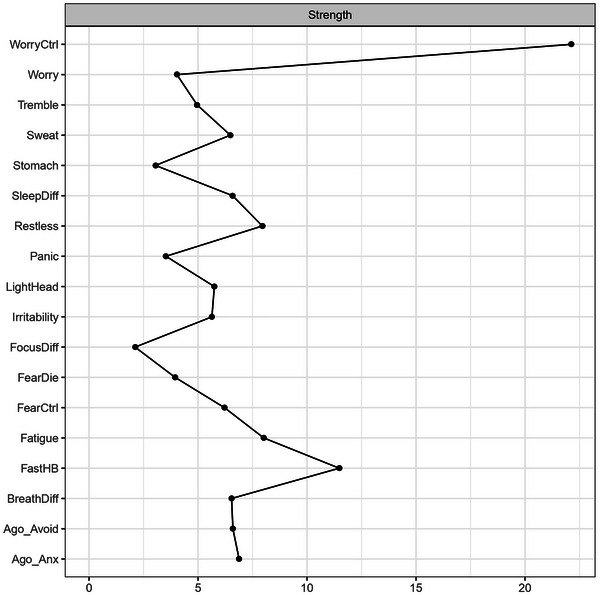
Strength centrality plot. Each point represents the strength centrality of an individual symptom node in the estimated network.

Strength centrality was computed to assess the relative importance of each symptom (Figure 2). Difficulty controlling worry (*WorryCtrl*) emerged as the most central node, underscoring its role as the key hub of the GAD subnetwork. *Restless*, *SleepDiff*, and *Irritability* followed closely, supporting their position as core features of chronic worry and hyperarousal in GAD (APA 2013). These symptoms collectively anchor the GAD structure and suggest that regulation and arousal‐related processes play a central role in the disorder's internal organization.

Within the panic–agoraphobia domain, physiological symptoms such as *Fast_HB*, *Sweat*, and *Tremble* showed moderate centrality. These symptoms were strongly interconnected within their own subnetwork but weakly linked to other domains, indicating that they function as localized hubs of panic‐related autonomic arousal rather than cross‐cutting features.

In contrast, *Ago_Anx* and *Ago_Avoid* were among the least central nodes, hinting to a possible self‐sustaining behavioral system, characterized by strong internal coupling and limited connections to other symptoms. Other peripheral symptoms, such as *FearDie*, *Stomach, FocusDiff*, and *LightHead*, showed limited centrality, indicating that although distressing, they contribute less to global network connectivity.

Overall, the centrality pattern underscores a dissociation between somatic‐arousal symptoms, which cluster locally, and cognitive‐affective symptoms, which function as the network's main drivers. This architecture supports the prominent role of worry‐related mechanisms in shaping the overall anxiety symptom structure.

### 3.3 Network Stability

Bootstrapped edge‐weights closely matched the original estimates and showed relatively narrow CIs (Figure S2), indicating good accuracy. Case‐dropping bootstraps yielded a strength centrality CS‐coefficient of 0.75, well above recommended thresholds for stability (Epskamp et al. 2018). Centrality difference tests confirmed that *WorryCtrl* was significantly more central than most other nodes, whereas differences among other highly ranked symptoms were less robust (Figure S3). Overall, the network showed satisfactory accuracy and stability.

Network stability and accuracy were assessed via nonparametric bootstrapping procedures. Our analysis revealed that bootstrapped edge‐weight closely matched the original estimates and showed relatively narrow CIs (Figure S2), indicating good accuracy.

The edge‐weight difference test showed that several of the strongest connections, such as those linking difficulty controlling worry (*WorryCtrl*) with restlessness (*Restless*), and Ago_*Avoid* with *Ago_Anx*, were significantly stronger than many other edges in the network. However, differences between some of the midrange edge weights, such as *Fast_HB–Trembling* and *BreathDiff–LightHead*, were not statistically significant, indicating that caution is needed when interpreting minor differences between moderate‐strength associations.

Stability of strength centrality was examined using case‐dropping bootstraps (Figure S3). Centrality scores remained highly correlated with the original network even when up to 75% of participants were randomly excluded, yielding a CS‐coefficient of 0.75, well above recommended thresholds for stability (Epskamp, Borsboom, et al. 2018).

The centrality difference test results confirmed that *WorryCtrl* was significantly more central than most other nodes in the network. However, other highly central symptoms, such as *Restless* and *SleepDiff*, did not significantly differ from each other or from some moderately central nodes (e.g., *Fatigue* or *Irritability*). Overall, the network showed satisfactory accuracy and stability.

## 4 Discussion

This study used network analysis applied to clinician‐administered diagnostic data (M.I.N.I.) to clarify the symptom‐level architecture of panic disorder, agoraphobia, and GAD in a large clinical sample. Beyond replicating established categorical distinctions, the results provide a structurally precise, data‐driven map of how these anxiety symptoms are internally organized. This combination of high‐quality diagnostic interviews and network modeling offers robust empirical support for a dimensional view of anxiety disorders, revealing patterns that traditional latent or categorical frameworks may obscure.

The network revealed two largely distinct clusters: one comprising panic–agoraphobia symptoms and another centered on GAD features. Within the panic cluster, autonomic arousal symptoms (e.g., racing heart, dyspnea, sweating, trembling, dizziness) formed a dense subnetwork, consistent with physiological models of panic disorder. Cognitive symptoms such as fear of losing control and fear of dying showed moderate links to these bodily sensations, supporting models in which catastrophic interpretations of somatic arousal create a self‐reinforcing cycle culminating in panic attacks (Margraf and Schneider 1995; Kyriakoulis and Kyrios 2023).

Notably, agoraphobia occupied a semi‐independent position within this cluster. Although avoidance and agoraphobic fear were tightly connected (White et al. 2006), their links to core panic symptoms were modest and centered on panic attacks rather than the full range of autonomic arousal. This suggests that, while agoraphobia often emerges in individuals with recurrent panic attacks, its behavioral architecture cannot be fully explained by panic symptoms alone (Wittchen et al. 2010). These results contribute new empirical evidence to the longstanding debate regarding the relation between panic and agoraphobia.

Biological models view agoraphobia as a consequence of recurrent spontaneous panic attacks (Klein and Gorman 1987), whereas cognitive–behavioral accounts describe it as a distinct disorder, maintained by anticipatory anxiety and avoidance learning (Marks 1987; Schmidt and Cromer 2008). Our findings align more closely with this latter perspective: panic attacks may trigger avoidance, but agoraphobic behavior appears to evolve into a self‐sustaining fear–avoidance system that persists independently. This interpretation is consistent with evidence that panic disorder and agoraphobia, though comorbid, occupy related but distinct positions within the anxiety spectrum (Greene and Eaton 2016). It also reinforces the need to target agoraphobic avoidance directly, even when panic symptoms have subsided (Holt and Lydiard 2007).

Turning to GAD, the second cluster emerged as a cohesive but asymmetrical structure anchored by difficulty controlling worry, which showed the highest centrality. This finding, in line with prior network research (Beard et al. 2016; Heeren et al. 2021), confirms that uncontrollable worry functions as the principal hub of generalized anxiety, shaping both cognitive and physiological manifestations. From this perspective, symptoms such as fatigue, irritability, restlessness, and sleep disturbance may reflect downstream effects of sustained cognitive tension. Consistent with this interpretation, these features were only weakly connected with one another in our network, despite their frequent clinical co‐occurrence. This limited interconnection contrasts with expectations that such arousal‐related features cluster tightly in GAD (Teed et al. 2022; Mishra and Varma 2023). A notable example is the fatigue–sleep disorders link, which appeared minimal in our network, contrary to the robust association typically reported in anxious and depressive populations (Ferentinos et al. 2009; McCAllum et al. 2019; Stanyte et al. 2024). This pattern suggests that arousal‐related symptoms in GAD represent parallel outcomes of chronic worry rather than a unified physiological mechanism. This nuance implies that interventions focused solely on hyperarousal or sleep–rest regulation may be insufficient without also addressing worry‐related cognitive processes.

The absence of direct edges between the panic–agoraphobia and GAD subnetworks further supports a structural distinction between fear‐based and worry‐based anxiety, suggesting that GAD functions as a self‐contained cognitive–affective network, driven by chronic worry rather than episodic fear like panic (Newman et al. 2013; Caldiroli et al. 2023). This dissociation echoes dual‐process models of anxiety (Lueken and Hahn 2016; Domínguez‐Pérez et al. 2025), where panic and agoraphobia reflect acute fear and avoidance processes centered on autonomic arousal, while GAD represents chronic anxious apprehension (Ohi et al. 2025). These structural patterns align with neurobiological evidence pointing to partially distinct neural circuits for fear (amygdala‐centered) and worry (cortical–striatal) processes (Domínguez‐Pérez et al. 2025).

The near absence of direct edges between panic–agoraphobia and GAD symptoms may appear at odds with the extensive literature documenting high diagnostic comorbidity among these disorders (Locke et al. 2015; Noyes 2001; Primiano et al. 2014). However, symptom‐level structural independence does not necessarily contradict diagnostic‐level comorbidity. Network models describe patterns of conditional symptom interactions, whereas comorbidity is defined by the co‐occurrence of diagnoses within individuals, reflecting distinct levels of explanation (Borsboom 2017). From this perspective, panic–agoraphobia and GAD may represent relatively autonomous symptom systems that are jointly expressed due to broader vulnerability factors, such as stable affective dispositions or generalized stress sensitivity, rather than through direct symptom‐to‐symptom reinforcement (Hankin et al. 2016). Moreover, mechanisms that cut across anxiety presentations but are not explicitly represented in the present symptom set, such as emotion dysregulation or chronic stress, may contribute to comorbidity without producing strong cross‐network edges.

At the same time, the M.I.N.I. does not allow for systematic quantification of psychiatric comorbidities across diagnostic categories. Although we provide an overview of diagnostic endorsement across M.I.N.I. dimensions to characterize the sample (Table 1), these data do not permit formal assessment of disorder‐level comorbidity. As a result, the present findings should be interpreted as symptom‐level associations rather than as evidence bearing on categorical diagnostic overlap.

The scope of these findings should also be considered in light of the heterogeneous clinical composition of the sample. Participants were recruited from a psychiatric outpatient setting for diagnostic evaluation, resulting in a population including individuals meeting full DSM‐IV criteria as well as those with subthreshold presentations. Although such heterogeneity may appear as a limitation in studies focused on categorical diagnoses, it reflects the clinical reality of outpatient psychiatric services, where patients frequently present with complex and overlapping symptom profiles that do not conform neatly to diagnostic boundaries. From a network‐analytic perspective, this variability represents a relevant feature, as network models are designed to capture patterns of symptom co‐occurrence across diverse clinical presentations.

Beyond these diagnostic constraints, aspects of the assessment strategy may have influenced symptom structure. Dichotomous M.I.N.I. items increase specificity but compress information about severity and functional impairment, potentially limiting sensitivity to weak or diffuse shared features. These factors may have contributed to the sparse connectivity observed between fear‐based and worry‐based symptoms. Future studies should incorporate severity and functional impairment sensible‐tools such as Likert intensity scales, the Sheehan Disability Scale (SDS) or the World Health Organization Disability Assessment Schedule (WHODAS 2.0) to provide a more comprehensive understanding of symptoms severity and the impact of anxiety disorders on participants' daily functioning.

Finally, the mode of assessment itself introduces additional methodological considerations. Although clinician‐administered interviews enhance precision and reduce self‐report bias (Bergelson et al. 2022; Rosenman et al. 2011), they also rely on a single source of information and may be influenced by rater judgment or interview structure. Future research would benefit from combining structured clinical interviews with dimensional self‐report measures and multi‐informant data, as well as from longitudinal designs that allow examination of dynamic symptom interactions over time. Such approaches may clarify whether the relative separation observed here reflects stable structural organization or measurement‐related constraints, and further elucidate the mechanisms underlying anxiety disorders (Taris et al. 2021; Verhulst and Neale 2021).

At the clinical level, our results underscore the importance of tailoring interventions to network‐specific mechanisms. For panic and agoraphobia, treatments targeting physiological arousal, interoceptive sensitivity, and avoidance behavior may disrupt the highly interconnected fear network. In contrast, GAD may require interventions aimed at cognitive flexibility, metacognitive awareness, and emotion regulation to weaken the internally coherent worry network (Domínguez‐Pérez et al. 2025).

## 5 Conclusions

This study provides detailed, quantitative evidence of the internal organization of panic disorder, agoraphobia, and GAD symptoms, showing that each forms a distinct yet partially interconnected system. Beyond confirming classical distinctions between fear‐based and worry‐based anxiety, our findings map the structural architecture of these domains: agoraphobia and avoidance form a behavioral subnetwork adjacent to panic, whereas GAD emerges as a cognitive–affective system centered on uncontrollable worry. These results refine understanding of anxiety comorbidity and support a network‐based, symptom‐level framework that informs more precise, mechanism‐targeted interventions.

## Author Contributions

**Emanuela Pizzolla**: conceptualization, methodology, formal analysis, data curation, visualization, writing – original draft, writing – review and editing. **Juan Martin Tecco**: conceptualization, data provision, writing – review and editing. **Moritz Bruno Petzold**: supervision, writing – review and editing. **Giovanni Briganti**: Conceptualization, supervision, methodology consultation, data analysis consultation, writing – review and editing.

## Funding

This work was supported by the AI4Brain project (grant number 747–UMONS_AI for Brain), funded by the Walloon Region (Service Public de Wallonie, FEDER) and coordinated by the University of Mons (UMONS), Belgium. The AI4Brain project integrates artificial intelligence and multimodal data analysis into the clinical management of psychiatric and neurological disorders.

## Ethics Statement

The authors assert that all procedures contributing to this work comply with the ethical standards of the relevant national and institutional committees on human experimentation and with the Helsinki Declaration of 1975, as revised in 2008. The study protocol was approved by the Ethical Committee of the CHP “Le Chêne aux Haies” (approval number 951, June 24, 2024).

## Consent

Because this study used fully anonymized archival clinical data, individual patient consent was waived in accordance with institutional and regional ethical guidelines.

## Conflicts of Interest

The authors declare no conflicts of interest.

## Supporting information




**Supplementary material**: brb371241‐sup‐0001‐SuppMat.docx

## Data Availability

The data supporting this study are not publicly available due to patient privacy and institutional data‐protection regulations. De‐identified data may be obtained from the corresponding author upon reasonable request.
