# Supplementary material for: A Network Analysis of Panic Disorder, Agoraphobia, and Generalized Anxiety Disorder in 463 Patients From a Psychiatric Hospital
Source: Brain Behav. 2026 Feb 10;16(2):e71241. doi: 10.1002/brb3.71241 (PMC12891977; doi:10.1002/brb3.71241)
Supplement: Supplementary file 1 — Supplementary material: brb371241‐sup‐0001‐SuppMat.docx [file BRB3-16-e71241-s001.docx]

# Supplementary material:

# Table 1s. Excluded Items with Justification

| Item Code | MINI Question | Rationale |
| --- | --- | --- |
| E2 | At any time in the past, did any of those spells or attacks come on unexpectedly or spontaneously, or occur in an unpredictable or unprovoked manner? | Conceptual overlap with E1 |
| E3 | Have you ever had one such attack followed by a month or more of persistent fear of having another attack? | Statistical and conceptual redundant with E6; not current |
| E4e | Did you have a choking sensation or a lump in your throat? | Conceptual redundant with E4d |
| E4f | Did you have chest pain, pressure or discomfort? | Conceptual redundant with E4a. Not very informative |
| E4i | Did things around you feel strange, unreal, detached or unfamiliar? | Low endorsement; statistically overlaps with E4g |
| E4l | Did you have tingling or numbness in parts of your body? | Low endorsement; Less specific; statistically overlaps with E4k |
| E4m | Did you have hot flashes or chills? | Low endorsement, less informative of the condition |
| O3b | Feel tense? | Conceptually overlaps O3a and statistically redundant with O3f |


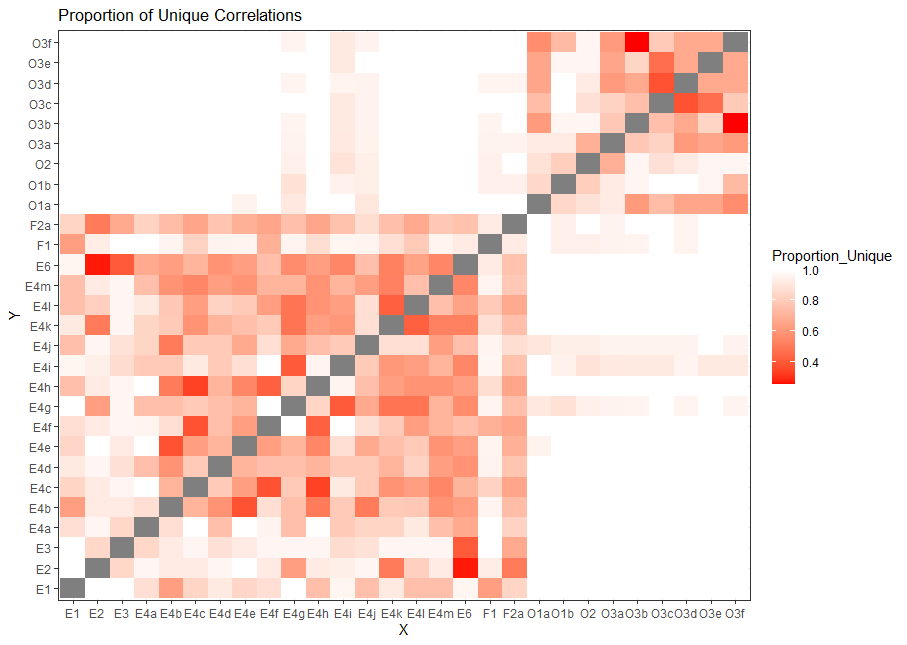


Figura S1. *Goldbricker plot showing the proportion of unique correlations among all candidate MINI items.*
This heatmap visualizes pairwise redundancy among 28 binary symptom items across the Panic Disorder, Agoraphobia, and Generalized Anxiety Disorder (GAD) modules. Darker red indicates a higher proportion of shared correlations between items, suggesting statistical redundancy (i.e., similar correlation profiles with other items in the network). Pairs with lower proportions of unique correlations (<0.25) were flagged as potentially redundant and reviewed for exclusion. Items along the diagonal represent self-correlation and are shaded in gray.


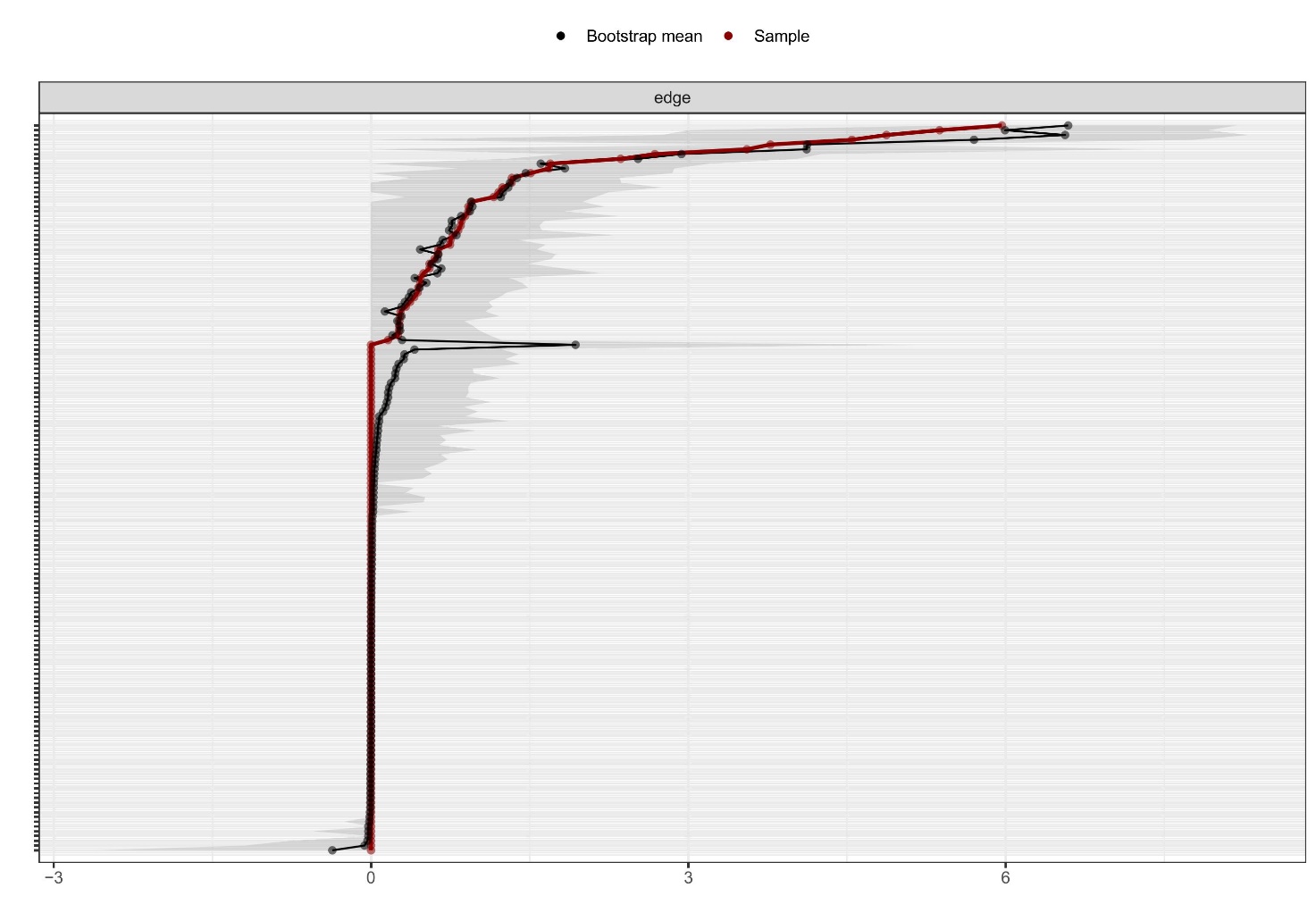


Figure S2. *Bootstrap estimates of edge weights.* The plot displays bootstrapped confidence intervals for all estimated edge weights in the network. The red dots indicate the original sample estimates, and black dots represent the average bootstrapped edge weights. The grey lines show 95% confidence intervals.


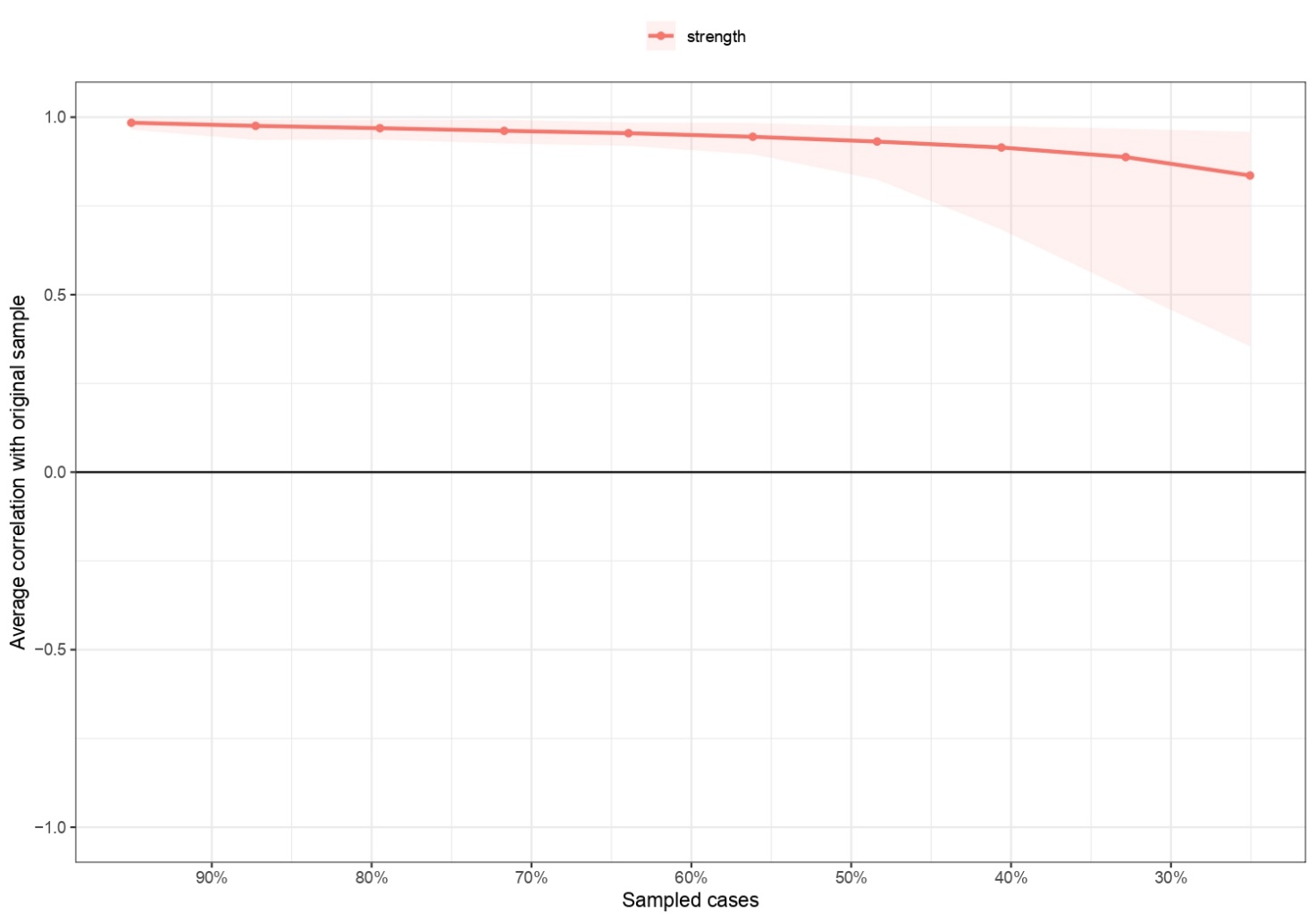


Figure S3. Case-dropping bootstrap analysis of strength centrality. This plot shows the average correlation between centrality estimates in the full sample and subsamples of decreasing size. The red line represents the mean correlation, and the shaded area denotes the 95% confidence interval.
